# A positive consequence of the COVID-19 pandemic: how the counterfactual experience of school closures is accelerating a multisectoral response to the treatment of neglected tropical diseases

**DOI:** 10.1098/rstb.2022.0282

**Published:** 2023-10-09

**Authors:** Donald A. P. Bundy, Linda Schultz, Manos Antoninis, Fatoumata B. M. Barry, Carmen Burbano, Kevin Croke, Lesley Drake, John Gyapong, Carol Karutu, Jimmy Kihara, Mouhamadou Moustapha Lo, Prerna Makkar, Charles Mwandawiro, Suzy J. Ossipow, Ana Ramos Bento, David Rollinson, Hemang Shah, Hugo C. Turner

**Affiliations:** ^1^ Research Consortium for School Health and Nutrition, London School of Hygiene and Tropical Medicine, London, WC1E 7HT, UK; ^2^ Global Education Monitoring Report, Paris, 75007, France; ^3^ World Bank, Washington, DC 20433, USA; ^4^ World Food Programme, Rome, 00148, Italy; ^5^ Harvard T.H. Chan School of Public Health, Boston, MA 02115, USA; ^6^ Imperial College London, London SW7 2BX, UK; ^7^ University of Health and Allied Sciences, PMB 31, Ho, Volta Region, Ghana; ^8^ END Fund, New York, NY 10016, USA; ^9^ KEMRI, Nairobi, 00200, Kenya; ^10^ World Bank, Yaoundé, Cameroon; ^11^ Health Compact, New Delhi, 122018, India; ^12^ Queensland Health, Brisbane, Queensland 4012, Australia; ^13^ Rockefeller Foundation, New York, NY 10018, USA; ^14^ Global Schistosomiasis Alliance, London SW7 5HD, UK; ^15^ CIFF, Delhi, 110030, India; ^16^ MRC Centre for Global Infectious Disease Analysis, School of Public Health, Imperial College London, London SW7 2BX, UK

**Keywords:** NTDs, deworming, London Declaration, COVID-19 pandemic recovery, school-based NTD programmes, School Meals Coalition

## Abstract

Global access to deworming treatment is one of the public health success stories of low-income countries in the twenty-first century. Parasitic worm infections are among the most ubiquitous chronic infections of humans, and early success with mass treatment programmes for these infections was the key catalyst for the neglected tropical disease (NTD) agenda. Since the launch of the ‘London Declaration’ in 2012, school-based deworming programmes have become the world's largest public health interventions. WHO estimates that by 2020, some 3.3 billion school-based drug treatments had been delivered. The success of this approach was brought to a dramatic halt in April 2020 when schools were closed worldwide in response to the COVID-19 pandemic. These closures immediately excluded 1.5 billion children not only from access to education but also from all school-based health services, including deworming. WHO Pulse surveys in 2021 identified NTD treatment as among the most negatively affected health interventions worldwide, second only to mental health interventions. In reaction, governments created a global Coalition with the twin aims of reopening schools and of rebuilding more resilient school-based health systems. Today, some 86 countries, comprising more than half the world's population, are delivering on this response, and school-based coverage of some key school-based programmes exceeds those from January 2020. This paper explores how science, and a combination of new policy and epidemiological perspectives that began in the 1980s, led to the exceptional growth in school-based NTD programmes after 2012, and are again driving new momentum in response to the COVID-19 pandemic.

This article is part of the theme issue ‘Challenges and opportunities in the fight against neglected tropical diseases: a decade from the London Declaration on NTDs’.

## 1. Introduction

Many human worm infections, such as soil-transmitted helminths (STH), appear to have been first acquired as a result of close contact with their original animal hosts when the latter were domesticated some 10 500 years ago, which helps explain their ubiquity as the most prevalent and widely distributed chronic infection of humans. Worm infections were among the earliest human infections to be recognized in early medical texts and putative treatments are known from the earliest pharmacopoeias [1]. In the twentieth century, community-based public health solutions were actively sought and piloted, but real success at scale has only been achieved in the past decade.

This is a success that has depended conceptually on major changes in the perception of the value of public health intervention versus individual treatment, on the availability of donated drugs, and on the cost-efficiency of targeted treatment campaigns outside of the traditional health system and delivered through schools. As a result, school-based mass drug administration (MDA) programmes emerged as one of the largest and most extensive public health interventions in low-income countries, with the National Deworming Day programme in India ranking among the most extensive regular public health interventions anywhere in the world [2].

Experience of the counterfactual of near-universal school closures during the COVID-19 pandemic has contributed to the recognition by countries worldwide of the importance of sustaining well-being in schools. The pandemic was recognized in January 2020, and by April 2020 almost every school in the world was physically closed to minimize the transmission of coronavirus infection. While some schools (generally in developed economies) could switch temporarily to online learning, this first-ever worldwide school closure excluded 1.5 billion children from formal access to education and generated the world's worst-ever education crisis, which remains a constraint on human capital development today.

This was the first time that schools had closed near-simultaneously worldwide, and provides a counterfactual experiment of not only the sudden loss of education facilities, but also the sudden withdrawal of school-based health services, such as deworming and other public health interventions. The World Food Programme (WFP) estimates that the closures resulted in the loss of daily school meals by 370 million children [3], for many the only reliable meal of the day. At the same time in 2020, 100 million people were driven into poverty, falling under the $1.90 threshold, especially in Africa. A Pulse survey by WHO in early 2021 determined that there was also a collapse in the coverage of MDA campaigns for neglected tropical diseases (NTDs), suggesting that after mental health NTDs were the most frequently affected public health service [4].

Governments and international development organizations have learned from this experience and created new organizations and new policies to help them reopen schools and establish new, improved and more resilient systems to promote health and well-being among schoolchildren and adolescents.

This paper explores why school-based deworming has become one of the most successful public health programmes of low-income countries in the twenty-first century, and why the experience of the school closures during the COVID-19 pandemic has led many countries worldwide to prioritize school-based deworming as part of their recovery efforts.

We dissect the scientific and policy foundations of school-based deworming that began in the 1980s and has continued since the COVID-19 pandemic. The first part examines why and how school-based MDA became such a success in the early twenty-first century after decades of stagnation. The second part examines why and how the counterfactual evidence from school closures, triggered by the pandemic, have increased the momentum of the response and opened up new and positive opportunities for the control and elimination of NTDs.

## 2. Part 1: Why and how school-based deworming programmes evolved to become among the most extensive school-based public health programmes globally

The Rockefeller Campaigns in some endemic countries and across the south of the USA were among the first MDA programmes [5,6]. The treatments used had poor efficacy and potential toxicity, but nevertheless analyses that have revisited these studies suggest that the programmes may have been effective. In creating human capital, Bleakley [7] reports effects for school children, although Roodman [8] offers a critical analysis [7,8] and undoubtedly influenced subsequent approaches.

**Figure 1.**
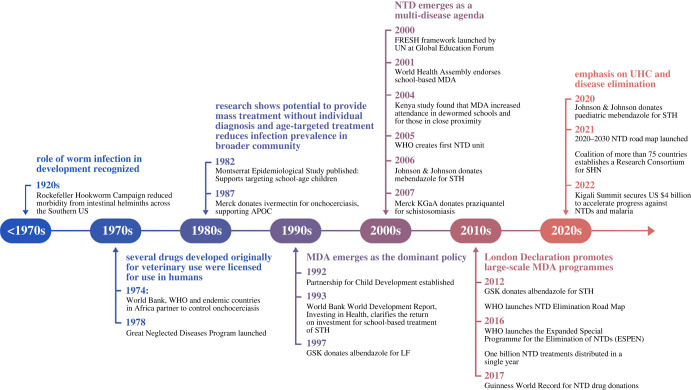
Timeline of key neglected tropical disease (NTD) programmes and policies through the early 2020s. APOC, African Programme for Onchocerciasis Control; FRESH, focusing resources on effective school health; GSK, GlaxoSmithKline; LF, lymphatic filariasis; SHN, school health and nutrition; UHC, universal health coverage. (Online version in colour.)

Efforts before 1970 were essentially large projects, while national programmes and a global movement emerged subsequently (figure 1). Several parallel developments can be seen here: evidence of impact at scale; a more cost-effective approach based on a stronger scientific under-pinning of biology, epidemiology and diagnosis that we might call today *implementation science*; and the protracted and challenging evolution of public health policy, aggravated by the ‘Worm Wars' controversy around the benefits of MDA in low prevalence settings.

### (a) The scale of the public health challenge due to worm infection

The Second World War resulted in worldwide migrations and movements of populations, including combatants, and the emergence of a new recognition of the importance of a global focus on health. Among chronic infections, worms were spotlighted as among the most ubiquitous, especially when sanitation broke down or was constrained by the fog of war. One of the most influential papers, entitled ‘This Wormy World' [9], highlighted how billions were infected worldwide, and, as illustrated by the dislocations of sanitation in war-time, everyone anywhere was potentially at risk. It was this concept that was taken up by Walsh & Warren [10] and addressed in the ‘Great Neglected Disease' programme launched by the Rockefeller Foundation in 1980. Even if each infection was not necessarily of public health concern, the accumulation of the burden across hundreds of millions of people most certainly was, a principle well illustrated by the epidemiology of *Ascaris lumbricoides* [11]. The World Bank's World Development Report of 1993, entitled ‘Investing in Health', explored the economic implications of this approach using new metrics, such as disability-adjusted life years (DALYs), to quantify the large-scale burdens. It Is notable that STH were selected as a key exemplar of this relationship, emphasizing the potential importance of lifelong educational attainment and its consequences for future earnings over the brief individual episodes of clinical disease [11].

### (b) Developing more efficient programmes

The increasing recognition of the public health importance of worm infections gave new momentum to mass deworming efforts [12]. There was an active repurposing of veterinary parasitic treatments for use in human infection during the 1970s. The safety profile and low cost of these drugs transformed the applicability and relevance of MDA, and these are today still the most commonly used treatments. Alongside the availability of these new tools there was also a step-change in understanding of the epidemiology of parasitic worms. This was driven by the application of population ecology to create a new field of parasite epidemiology, based on the principles of whole-organism population dynamics [13–17]. A key new insight from this approach was that the worm burden—the number of adult worms present in an infected host—was density-dependent and thus both morbidity and transmission were scaled by the number of worms present, and so both would decline with treatment that expelled adult worms and reduced the size of the worm population [18–21]. Infection intensity is typically host age-related, with the highest intensity of infection for roundworm, whipworm and schistosomes in school-age children. Hence a disproportionate reduction in disease should result from treating school children since they are the most intensely infected age group in the population. The application of these theoretical relationships to the design of actual programmes showed that a disproportionate reduction in infection across the whole population, including uninfected adults, could be achieved by treating only the school-age children [12,22]. Since these children are the main source of infection to the rest of the population, reducing their worm burdens also reduced the number of infectious parasite eggs entering the environment, and so reduced exposure to infection of the population as a whole. This finding in turn led to the recognition that ‘externalities', essentially free benefits, could be achieved by age-targeting, with important implications for cost-effectiveness [23].

Other contributions to cost-effectiveness were achieved through a surge of implementation science around the design of targeting. For example, one policy game-changer arose from the recognition that treatment was around a tenth of the cost of individual diagnosis, so that if a population had already been identified as having infections levels that justified treatment then MDA could proceed without further need for individual diagnostics [12], although this assumption does of course change once prevalence has been significantly reduced by a successful programme and different assumptions are required for the end game.

### (c) Controversy in assessing impact of deworming: the ‘worm wars' and beyond

As deworming emerged as a global public health response there was a parallel increase in scrutiny and debate, which sometimes overflowed beyond science, with entrenched positions adopted by journals, their editors and the social media. Here, we comment on three aspects: the statistical evidence from meta-analyses; the long-term impacts; and the working consensus that has emerged.

Perhaps the most salient debate was around how to aggregate and interpret some forty years of MDA randomized control trials (RCTs), many of which were underpowered, hence the emphasis on meta-analysis. A series of Cochrane systematic reviews and meta-analyses, mostly recently in 2015 [24] and in 2019 [25], and a Campbell Collaboration review [26] found no average impact of MDA for deworming on nutritional and educational outcomes, while a larger aggregation from the same pool of studies found significant effects, most notably for weight gain [27]. As the debate continued, however, the point estimates for the included studies converged, such that the most recent Cochrane review [25] estimates an average effect of MDA on weight of 0.11 kg (95% confidence interval (CI) −0.01, 0.24) while a different but contemporaneous review [27] estimates an effect of 0.13 kg (95% CI 0.03, 0.24). There is general agreement that trials which dewormed only children with confirmed worm infections found large benefits. MDA is given to the whole population, whether infected or not, and the average outcomes reflect the impact on both infected and uninfected individuals.

A separate literature assessed long-term benefits to health and broader socioeconomic outcomes over the life course. Debate on the meta-analyses spilled over to interpretation of a particularly influential study by Miguel and Kremer [23] of school-based deworming in western Kenya and the longitudinal tracking of the trial population [28,29]. In a setting where STH infection was almost universal, mass deworming increased attendance at dewormed schools and had external benefits for those living in close proximity, with some controversy around the geographical distance over which beneficial effects were detected [30]. A decade after treatment, dewormed men worked 17% more hours per week, and dewormed women were more likely to pass exams and attend secondary school [28]. After 20 years, the more intensively treated groups had *per capita* household consumption expenditure that was 14% higher (*p* = 0.06) [29], suggesting a 37% annual social internal rate of return for MDA. Detailed scrutiny of the longitudinal studies [31,32] has largely supported the validity of these findings. But longitudinal studies in schools with low to medium prevalence and low intensity of infection did not show an impact of MDA in rural China [33] or in rural eastern Uganda [34], and it has been suggested that the high intensity of infection around Lake Victoria when the Kenya study was begun in 1998 could explain the large life-course gains in that setting.

This complex body of evidence has presented a challenge for policy makers who must take into account expected benefit, cost and equity in deciding whether MDA can be expected to be cost-effective relative to other health interventions in a given setting. What has emerged is a consensus, based on clinical evidence and the meta-analyses, that *infected* children should be treated [35]. Since treatment of infected children is uncontroversial, and treatment is deemed safe, it has also been taken as uncontroversial to presumptively treat high prevalence populations with MDA. The policy question at a global scale is where to place the threshold for treatment: WHO guidelines place the threshold for annual deworming at 20% prevalence [36], and a recent modelling study [37] supports this threshold.

### (d) Towards a new global policy on deworming

Interest in controlling helminth infections surged after the decision by Merck in 1987 to donate the newly discovered veterinary drug ivermectin for use in controlling river blindness, and pledging ‘as much as necessary for as long as necessary' [38]. In 1974, an agreement by then World Bank President, Robert McNamara, with the WHO, development partners, pharmaceutical companies and African governments helped establish a partnership to control river blindness (APOC). This neglected tropical disease (NTD) investment also represented the first World Bank health project [39]. This led to further donations: from GlaxoSmithKline in 1997 and again in 2020 for STH; from Johnson & Johnson in 2006; and from Merck KGaA in 2007 for schistosomiasis, as shown in table 1 [40]. The FRESH (Focusing Resources on Effective School Health) framework was the first education sector adoption of the MDA approach, launched at the World Education Forum in Dakar in 2000 [44], and was reinforced in 2001 by a World Health Organization (WHO) declaration in support of school-based MDA [45].

**Table 1.** Medicines donated by pharmaceutical companies to the World Health Organization for the control of PC-NTDs.

company	drug donated	susceptible disease	commitment
Merck & Co	ivermectin (Mectizan)	onchocerciasis and lymphatic filariasis	Since 1987: unlimited supply until onchocerciasis is eliminated
			Since 1997: unlimited supply until lymphatic filariasis is eliminated from Yemen and African continent in regions where lymphatic filariasis is co-endemic with onchocerciasis
			2018–2025: up to 100 million treatments to eliminate lymphatic filariasis using WHO-recommended triple-therapy MDA in regions that are not co-endemic for onchocerciasis
GlaxoSmithKlein (GSK)	albendazole	lymphatic filariasis	Since 1997: up to 600 million tablets annually until lymphatic filariasis is eliminated as a public health problem
		STH	2012–2020: 400 million tablets annually for the treatment of STH in school-age children
Pfizer	azithromycin	trachoma	1998–2025: unlimited quantity to eliminate trachoma as a public health problem
Johnson & Johnson	mebendazole	STH	2006–2025: initially 50 million annual donation, revised to 200 million annual donation in 2010, for the treatment of STH in school-age children. From 2020, Johnson & Johnson is donating its chewable paediatric formulation, which can be safely used by preschool-age children.
Merck KGaA	praziquantel	schistosomiasis	Since 2007: initially up to 20 million tablets to treat schistosomiasis in school-age children; commitment revised to 250 million tablet donation in 2012, until schistosomiasis is eliminated as a public health problem

Source: Published in Bundy *et al*. [40,41], and adapted from Bradley *et al*. [42] with additional information from Johnson & Johnson [43].

By the start of the second millennium there was already an established approach to community control of some of the most ubiquitous chronic infections of low-resourced communities. What happened next was that these new approaches to epidemiology, economics and policy began to be syncretized into a new and popular ‘branding' for these ancient diseases.

### (e) The new ‘brand': neglected tropical diseases

Governments, often with the support of private sector donations, intensified their efforts to address ubiquitous diseases that affected the development of their children in middle childhood and adolescence. In particular, they built on the WHO policies in support of school-based MDA, and access to private sector donations. The 'great neglected diseases' programme [46] launched more than 20 years previously laid the foundations for this new perspective, and in the mid-2000s this was rebranded as NTD [47–50]. A new NTD department was opened at WHO in 2005, and during this period some, but not all, endemic countries were able to conduct MDA programmes. It is worth noting that the time scale of this translation from good idea to actual programme response is a good fit with the estimates of 13–17 years made by many implementation science investigations [51–53].

‘The London Declaration’ by development partners in 2012 created a coalition in support of the WHO 2012–2020 'road map' [29] and provided a stronger platform to continue and expand drug donations. Fourteen billion treatments to eliminate ten NTDs were pledged by thirteen pharmaceutical companies over a ten year time horizon (table 1) [54]. This donation was valued at US $18 billion, and was a response to the constrained fiscal capacity of governments in those countries where NTDs were most prevalent, and it attracted further support to help countries deliver these treatments at scale [55]. As a result, more than a billion treatments annually were being delivered worldwide by 2016 [56], and by the following year over 200 million donated doses were arriving at drug distribution facilities across six countries: a new Guinness World Record for the largest drug donation in a single day [57].

As described in the next section, prolonged school closures to limit the transmission of COVID-19 provided counterfactual evidence to the importance of the school platform in achieving disease control targets; WHO Pulse surveys (see https://www.who.int/teams/integrated-health-services/monitoring-health-services/global-pulse-survey-on-continuity-of-essential-health-services-during-the-covid-19-pandemic) indicate that NTD programs were among the most affected health programs globally.

Figure 1 shows that it took a combination of new approaches to launch the ultimately successful movement towards making deworming universally accessible.

### (f) Experience of school-based mass chemotherapy as a community intervention

WHO estimates that 3.3 billion treatments for STH infection have been delivered through schools since 2010 [58]. In the period between 2010 and 2015, the number of children living with these infections may have fallen by half [59]. In endemic countries, more than 60% of school children were reached, with 28 (of 96) countries sustaining treatment beyond 5 years [58]. Among these countries, Burkina Faso and Mali have since stopped MDA and have begun regular surveillance to guard against resurgence [58]. Similarly, analysis of data from 2000 to 2019 suggests that schistosomiasis prevalence in school-aged children has decreased considerably, and several countries (e.g. Burundi, Eritrea, Eswatini, Gambia, Lesotho and Rwanda) are in a position to consider elimination strategies in line with the WHO NTD Roadmap goals [60].

There are important successes. In Kenya, the National School-Based Programme includes extensive monitoring and evaluation to track annual and overall impact [61]. In India, the National Deworming Day is the world's largest single-day public health campaign, covering over 200 million children [2]. With substantial government investment and political commitment, India is the exemplar of domestic contribution to deworming, and also of implementing allied programmes, such as its Total Sanitation Campaign. Reassessments have enabled India to conceptualise a 5-year Road Map, further optimizing domestic deworming investment.

The roll-out of school-based MDA programmes as part of the NTD efforts has provided some important insights into the value of this approach. MDA campaigns for STH and schistosomiasis are largely conducted through schools, reflecting the feasibility and cost-effectiveness of using existing infrastructure to reach this cohort during some of their most vulnerable years and the near universality of children enrolled in primary school.

It is important to note that while the out-of-school rate among primary school-age children has halved globally from 19% in 2000 to 9% in 2020, universal enrolment has not yet been achieved [62]. According to a new cohort-based estimation model, the out-of-school rate in low-income countries only fell from 26% to 20% over this period. In sub-Saharan Africa, it increased in a quarter of countries between 2015 and 2020, while the out-of-school population increased from 33 million in 2010 to 36 million in 2020 among those of primary school age (and from 78 million to 96 million among those of primary and secondary school age) [63]. One-in-four children do not complete primary school in the region. In other words, the goal of universal primary education that was first set for 1980 remains elusive for the region whose children are most vulnerable to NTDs.

Schools represent a cost-effective and efficient platform through which to deliver an essential integrated package of health and nutrition services that goes beyond deworming to address school meals, water, sanitation and hygiene (WASH), menstrual health and some of the other common issues that emerge during school-age and adolescence [64]. Recognizing the importance of health interventions in learning, the Global Partnership for Education republished the Disease Control Priorities, Third Edition volume, ‘Child and Adolescent Health and Development' for an education audience [65].

### (g) The concept of the next 7000 days

Health investments in children have tended to focus on the first 1000 days of life, spanning conception to two years of age. Education investments largely cover the period from 5 years of age to the early twenties; increasingly designated as the ‘next 7000 days' [66]. As evidence has accumulated it has become increasingly clear that there is a need for continuing investment from the first 1000 days through the next 7000 days to respond to development vulnerabilities in these later years, especially around puberty and brain development, and to build upon and sustain the gains of early intervention [67].

Educational achievement during the next 7000 days depends on both good education and the well-being of the school child, and is a crucial element of human capital creation [68]. Schools and the education system provide an existing platform to deliver interventions for many of the common causes of morbidity that arise in school children and adolescents [69,70]. Deworming, for example, can improve both current health and long-term outcomes as an adult. A study of poverty alleviation in Kenya shows that those children who had access to deworming drugs at school were later able to earn higher incomes (13%), achieve greater consumer spending (14%) and had greater likelihood of obtaining jobs with higher wages and better career opportunities [29].

#### (i) Looking towards the endpoint

Sustained school-based treatment can control morbidity from moderate to heavy infection, but on its own may not prevent reinfection. The impact is strengthened by combining it with WASH and health education [20], though these are often omitted from NTD programmes [71]. Good sanitation and hygiene are necessary to sustain effects [72].

These observations support the conclusion that there is unlikely to be an ‘endpoint' that can be achieved by deworming alone, although there are trials that deserve further analysis to provide greater insights into whether morbidity control is likely to support an eradication paradigm in the long term: the Tumikia RCT, comparing school- and community-based interventions [73]; the WASH Benefits RCT in Kenya and Bangladesh [74,75]; and the multi-country Deworm3 RCT [76].

That an endpoint may be an elusive goal through treatment alone does not mean that treatment is likely the main lever towards eradication. There may also be lessons from nations such as Japan and South Korea that have achieved eradication through a treatment-led process [77,78]. In these countries, the drug programmes were supported by the socioeconomic improvements that flowed from economic development. We need to better understand whether these outcomes are likely in contemporary society, perhaps by tracking whether the combination of economic growth and targeted treatment programmes in countries with successful, active deworming programs, such as India and Kenya, is likely to have similar positive outcomes to those documented in Japan and Korea.

### (h) Human capital development and the importance of investing in schoolchildren and adolescents

Human capital, which is defined by the World Bank as the sum of a population's health, skills, knowledge and experience, is a key contribution to the competitiveness of a country in today's world. The people make a major contribution to the wealth of their nation. In low-income countries that contribution may be around 30–40% of the nation's wealth, but for high-income countries that proportion can reach 70–80%—which can strengthen a country's competitiveness. Some 10–30% of *per capita* income reflects human capital, and the gross domestic product in African countries could be 2.5 times higher than it is today if the health and education systems delivered on the need for human capital creation [79].

Well-being, health and education are the foundations for growth and economic development [80]. The economic trajectory of most countries where STH are present today is also trending upwards, and the World Bank estimates that a third of low-income countries in Africa will be middle-income countries by 2030.

## 3. Part 2: How the counterfactual evidence from the worldwide closure of schools during the COVID-19 pandemic has strengthened the resolve for countries to integrate school-based NTD programmes in their investment in the education and well-being of children

School closures in response to the COVID-19 pandemic have highlighted the vital role that schools play in protecting the health and well-being of learners. It has provided the counterfactual evidence of what happens when school-based health services, including deworming, are no longer provided. This experience has strengthened countries' resolve to integrate school-based NTD programmes in their investment in the education and well-being of children, and to create a global Coalition to help make that happen.

In January 2020, around a billion people a year benefitted from NTD campaigns worldwide, the majority being school-age children and adolescents receiving school-based MDA for worm infections. In most cases, these programmes were dependent upon pharmaceutical company donations of drugs. Also in January 2020, it is estimated that 388 million children received daily meals at school (equivalent to some 69 840 billion meals in total) [81]. In total, 98% of these meals were provided by governments using their own domestic funds, totaling a US$ 43 billion investment by governments globally. In contrast, overseas development assistance (ODA) investment is of the order of US$ 300 million annually [3]. This huge level of domestic support is a clear indicator of the value that governments placed on the current health and well-being of their children and adolescents as an investment in their future contribution to human capital [41].

These investments in the next generation all came to a shuddering halt around April 2020, when nearly every country in the world announced its intention to close its schools and require all students to remain at home to minimize the transmission of coronavirus contributing to the COVID-19 pandemic. It is perhaps ironic that the health needs of the children themselves, whose health was little affected by coronavirus infection, was not the main concern of that policy decision, but rather was in recognition of their role in transmission of the virus to multiple generations of family members [82].

It is estimated that 1.5 billion children were immediately deprived of access to school. While some children in some high- and middle-income countries continued education through distance education tools, principally digital, a majority did not. It is estimated that less than 10% of children in Africa had access to digital sources for distance education [83]. In some countries schools re-opened relatively quickly, for many there was a disruptive cycle of re-opening and re-closing, and for some, this withdrawal of schooling was long-term: for example, schools in Uganda and the Philippines only reopened more than two years later. This was the first time that so many schools worldwide had synchronized closure; this had not happened on this scale even during the World Wars, and it resulted in the world's worst education crisis ever. In high-income countries it is now recognized that generations of children have lost out on educational opportunities that will have lifelong consequences, while in low-income countries the World Bank estimates that the already very poor level of educational achievement has deteriorated further: the proportion of children in Africa able to read a simple age-appropriate sentence has declined from 70% to 53% [84].

In addition to the education impacts, there were consequences for health and well-being. At the height of the pandemic, an estimated 370 million children in 161 countries lost access to what was for many their only dependable meal of the day [85]. In parallel, an additional 100 million people were pushed below the $1.90 poverty threshold in 2020, primarily concentrated in the Africa region [86]. In addition to these negative schooling outcomes, the COVID-19 pandemic also had negative sexual and reproductive health consequences. In Kenya, for example, girls had twice the risk of falling pregnant prior to completing secondary school and three times the risk of school dropout [87]. A Pulse survey by WHO in early 2021 determined that there was also a collapse in the coverage of MDA campaigns for neglected tropical diseases (NTDs), suggesting that after mental health NTDs were the most frequently affected public health service [4].

The experience of having service delivery interrupted by prolonged school closures, and the inadequacy of most health systems in serving school-age children and adolescents, have together increased the resolve of government public health actors to use schools to implement public health initiatives to reach large numbers of children and ensure these students can participate in school effectively as healthy learners. MDA in school settings responds to the needs of the learner [88] and, especially if linked with health and WASH interventions and infrastructure [89,90], can contribute to bridging gendered disparities, especially for adolescent girls in the context of menstrual health [91]. This has resulted in responses from national governments and non-state actors.

### (a) The role of national governments: the school meals coalition

**Figure 2.**
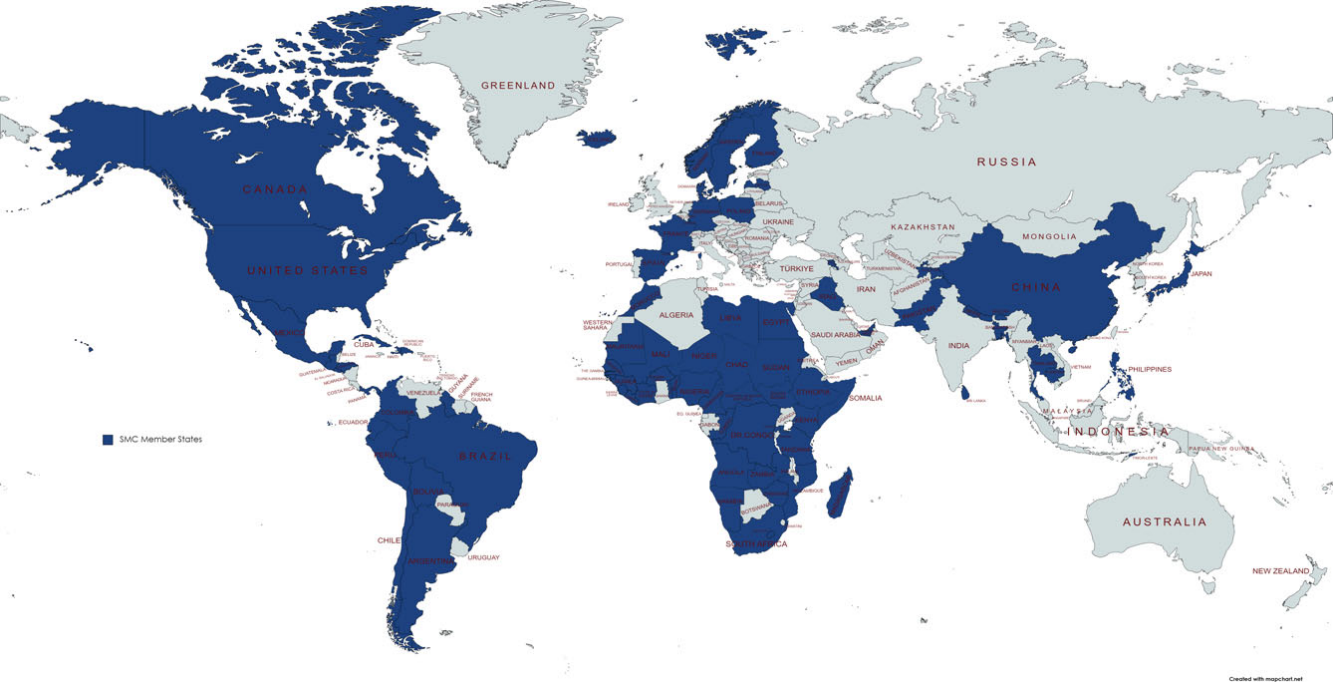
Eighty-six countries have signed the Declaration of Commitment to the School Meals Coalition as of July 2023. (Online version in colour.)

The school closures and the ensuing social consequences, which were experienced in countries at all levels of economic development, led to national political leaders forming a Coalition at the 2021 UN Food System Summit with the specific aims of rebuilding the school-based services that had been severely damaged by the pandemic closures and ensuring the wellbeing of current and future generations of school children. Today, some 86 countries, represented at the highest levels of government, have created a ‘School Meals Coalition' with three specific goals: (i) to restore national school meals and complementary school health programs to pre-pandemic coverage by 2023; (ii) to develop new approaches to reach an additional 73 million of the most in-need children who had not previously been reached, by 2030; and (iii) raise the quality of school health and nutrition programmes globally by 2030 [92] (figure 2). As a measure of the efficacy and energy of this Coalition, it is notable that goal (i), established in 2021, seems to have been achieved by the end of 2022 (see §3c below). In addition, the WFP, the largest UN organization and the largest humanitarian organization, has identified deworming and school-based interventions as a priority in its 2020–2030 strategy [93].

Recognizing the fundamental importance of good science to help countries achieve these goals, the Coalition has created a Research Consortium for School Health and Nutrition to provide independent and credible evidence on good practice (www.lshtm.ac.uk/shn). The Research Consortium has identified the need to provide policymakers and parliamentarians with the evidence on complementary school-based health interventions, including deworming, alongside other key health, nutrition, and well-being interventions that can be cost-effectively delivered through the education system [94].

### (b) The role of international non-state and intergovernmental organizations

In addition to supporting government, the Coalition is also working with non-state actors to help provide complementary school-based health interventions.

The World Bank has been actively engaged in financing community-based NTD control and elimination since the 1970s and has been instrumental in financing school health and nutrition programmes in many countries across the world for the past two decades. In 2017, with the launch of the World Bank's Deworming Africa Initiative (DAI) in partnership with the Bill and Melinda Gates Foundation, the World Bank has increased domestic financing for deworming (increasing annual investments from US$ 3 million to US$ 14 million between 2013 and 2018 [95]) and supported many countries with the integration of deworming in essential health packages for primary healthcare and school health packages. More recently, the World Bank has been a leader in financing NTD programs in countries where resources have shifted due to the COVID-19 pandemic [96].

The End Fund is one of the actors that are spearheading this movement. The END Fund, already working with 33 countries, seeks to raise new sources of philanthropic capital for the NTD community, clarify messaging around NTD treatment effects, and to support domestic ownership. Domestic ownership means that dedicated government and civil society organizations lead the pathway to elimination, and ensure sustainability in the long run. With this in mind, the END Fund is steadfast in bringing new domestic actors to the table and ensuring they have the necessary resources to get this essential work done. With the ethos of ‘leaving no one behind', the END Fund strengthens school-based health interventions as well as community-directed interventions while also supporting innovative approaches that monitor the impact of treatments, strengthen countries' health systems and surveillance capacity, and ensure that no segment of the population or country is left behind because of their gender, ethnic identity, geographical location or other factor [97]. The END Fund also hosts the $100 million multi-donor Deworming Innovation Fund to target the interruption of transmission of soil-transmitted helminths and schistosomiasis and to serve as an innovation hub for testing and documenting novel and sustainable approaches to reach this goal. This fund targets disease elimination in four countries: Ethiopia, Kenya, Rwanda and Zimbabwe. Today, more development partners and governments are thinking beyond ‘business as usual' approaches to enable the sector to achieve the UN Sustainable Development Goal (SDG) aspirations for 2030.

Similarly, the Global Schistosomiasis Alliance (GSA) is involved with a wide range of stakeholders to strengthen school- and community-based schistosomiasis control and elimination efforts. In 2020, estimates suggest that at least 241.3 million people in 80 countries—the vast majority in sub-Saharan Africa—required treatment for schistosomiasis [98]. Merck KGaA have been donating the drug praziquantel for oral treatment of the disease (table 1) and will provide up to 250 million tablets per year, approximately sufficient to treat 100 million school children, until elimination as a public health problem has been achieved. The most effective and cost-efficient approach to reach children in need has been to deliver medicines through the school platform. Treatment with praziquantel is safe and efficacious but it is best taken with food to minimize any side reactions. Regular treatments can reduce prevalence and intensity of schistosomiasis, but to move to elimination of this water-borne disease other interventions must be introduced including safe water and sanitation, behaviour changes and integrated vector control; schools can assist again by ensuring that children better understand actions needed to avoid infection. Many mass treatment campaigns for schistosomiasis were severely disrupted due to COVID-19, potentially leading to an increase in infection and a requirement to restart programmes quickly [99]. There is no clear evidence that the interruption of treatment had immediate consequences for the health of endemic populations, but effects on morbidity may take years to emerge. Initial data suggest that mass treatment for STH has recovered more quickly than for schistosomiasis [99,100]. One encouraging development in the pipeline is a paediatric formulation of praziquantel targeting children aged three months to 6 years, which will be of huge benefit to this previously neglected age group.

The WHO NTD roadmap (2021–2030) outlines three pillars to support efforts to control and eliminate NTDs, and the third pillar concerns changing operating models and culture to facilitate country ownership [101]. Non-state actors have a key role to play in this. For example, the GSA identifies the growing momentum embedded within the School Meals Coalition to accelerate health and nutrition in schools as an opportunity to develop better links between different sectors and ministries, and so to facilitate increased country involvement in the delivery of much-needed medicines to treat both schistosomiasis and the soil transmitted helminths in school-age children [102].

### (c) Emerging evidence for the rebuilding of school-based health services

The WFP biennial report ‘The State of School Feeding Worldwide 2022' has just been published [3]. The primary purpose of this report is to provide an update every two years on global progress with school meals programmes, and to help the 86 countries in the School Meals Coalition to track their progress. This tracking role includes school-based health services, and the report is the only regular source of data on school health programming globally. The 2022 report used its surveys to estimate that the number of children receiving daily school meals was 388 million in January 2020, and that a majority of these programmes also delivered school-based deworming. The 2022 report also provided data suggesting that the number of children fed had fallen to less than 20 million by April 2020, based on an estimate that 370 million children had lost their access to a school meal during the peak of school closures. The latest report suggests striking progress since then, and now estimates that the number of children receiving school meals in 2022 has rebounded to 418 million, up 40 million on pre-COVID-19 levels. It also reports that a majority of these programmes also deliver complementary school-based deworming.

This has two important implications. First, it suggests that coverage of school meal programmes provides some proxy for deworming coverage. School meal programmes are massively more complex and expensive than deworming programmes; for example, average annual per person costs of school meals is in excess of US$ 50 per child per year, while the equivalent for deworming is circa 50 cents [64], so it is not surprising that countries that can afford the former would likely leverage the health benefits of the latter. Secondly, it suggests that the governments of the Coalition have indeed focussed on achieving their first declared goal (to restore what they had by 2023) and have likely already achieved that goal in a majority of countries.

It is already apparent that the School Meals Coalition is providing new momentum for rebuilding school-based health services, and for the school-based delivery of NTD treatments in particular. This bodes well for a continuing and robust future contribution to NTD goals. This is particularly impressive because these programmes continue to be more than 90% supported by domestic funds despite the severe constraints on fiscal space occasioned by the COVID-19 pandemic.

### (d) Looking beyond the school-based approach

The renewed success of school-based MDA will be a continuing key contributor to the WHO SDG-linked goals for NTDs in 2030, but it cannot be the whole solution.

Free or subsidized MDA is a move towards essential health service provision [103], but viewing this as a proxy for universal health coverage (UHC) masks both the current inequities in provision and the lack of patient control over treatment options [104]. Providing on-demand medicines through primary healthcare facilities raises challenges, including willingness-to-pay for preventative treatment and capacity of health systems to pay for diagnostics [105]. It is worth reflecting on Japan, which did precisely that: basing its successful national deworming strategy on a patient-led pay-for-test-and-treat approach targeted at school children. These challenges could be addressed by including deworming within an essential package of services financed by national governments [106].

If treatment were to become available in primary healthcare facilities there would need to be campaigns to raise awareness, explaining the principles of UHC and the availability of treatment [107]. There is preliminary evidence of demand for preventive chemotherapies outside of MDA in Bangladesh, where school-aged children only were targeted for treatment and yet adults were found to experience a similar decline in prevalence when compared to treated children over a 10-year period [108]. The authors speculate that this decline may be due to adults actively purchasing deworming medicines or improved WASH. There are other examples where child-health days, women's reproductive health clinics and vaccine campaigns have been employed to raise patient-led demand.

Combining school-based treatment with care-seeking from government health centres is becoming part of the way forward but is not without challenges. Socio-economic status differs between those who seek treatment and those that don't, or who favour private clinics or traditional healers [109]. Those who most need treatment may be those least likely to ask for it, which suggests a need for better understanding of the potential role for community advocates for on-demand treatment [110].

#### (i) Areas that warrant continuing attention

School-based MDA has been adopted by nearly all countries where STH infection is endemic at levels considered to be of public health consequence and has been the mainstay of schistosomiasis control programmes throughout Africa. Many of challenges were overcome in this process, but there are four potential barriers to progress that warrant continuing attention.

First, it is clear that it has taken a really long time to roll out this seemingly very simple intervention, although the time-scale accords with current implementation science concepts [51–53] that suggest it takes some 13–17 years to translate from research to action. In the case of NTDs the concept of cost-effective mass treatment emerged in the early 1990s, was followed by the emergence of the NTD agenda in the early 2000s, followed by the practical roll-out from 2012 onwards. This suggest that if we anticipate any change by 2030 we are already too late with the next big idea.

Secondly, and perhaps most worryingly, the main pharmaceuticals in use are based on products first discovered for veterinary applications, and there has been no break-through deworming drug for human, or indeed veterinary use in the last 30 years. This means that there are no back-up options if resistance becomes an issue.

Thirdly, there has been limited progress made towards diagnosis of both STH and schistosomiasis at mass scale in areas of low prevalence, and thereby limited guidance that country governments can use to establish robust surveillance systems. This will become even more necessary with decreasing prevalence as we approach the stated goal of the 2021 WHO Road Map of elimination of STH and schistosomiasis as a public health problem.

Lastly, climate change may significantly alter the geography of disease, with the potential for a wider occurrence of disease in temperate regions. MDA programmes will need to pay attention and respond to potential changes in the distribution and transmission patterns resulting from global climate change and soil conditions [111].

## 4. Conclusion

For thousands of years, we have been aware of worm infections of humans, and the ill-health they cause, but it is only in the last 5–10 years that the world has made significant progress in controlling these infections as a public health problem.

The history of this success story reflects a slow, decades-long science-driven process which, for the most ubiquitous infections led to adoption of school-based delivery of treatment at global scale. The scientific process began when simple, safe and effective treatments were first developed some 40 years ago. Then new practical and cost-effective ways of delivering these drugs in the community emerged with the revolutionary ecological understanding of the epidemiology of worms in the 1980s and 1990s. The pharmaceutical industry recognized the potential scale of benefit from an approach based on this new-generation, evidence-based mass treatment and offered support to the poorest countries on an unprecedented scale of hundreds of millions of treatments. These components came together in the new millennium, driving the policy change and national commitments that led to the global call to control NTDs and resulted, for the first time ever, in the regular successful treatment of over a billion people annually.

The future of these already very successful programmes will likely be shaped by two recent changes in policy direction. On the one hand the WHO roadmap for NTDs, that sets out global NTD policy from 2021 to 2030, and on the other hand the rise of country-led school health and nutrition programmes, as nations re-open schools and recover from the COVID-19 pandemic. The former calls for increasing transition to country leadership of national NTD programmes. The latter is already a country-led movement, the School Meals Coalition, and prioritizes rebuilding school-based delivery systems to promote the well-being of school children and adolescents. Their objectives are compatible: as the world's health organization the WHO approach is, appropriately, focused on improving health, while the 86 countries that make up the Coalition pursue a more comprehensive goal that includes not only health, but also the well-being, education and human capital that will help secure the economic future of the participating nations.

As the world moves towards 2030 there are now new players driving deworming. Their challenge is to emphasize their compatibility and minimize their differences, and to support the transition towards stronger national leadership. An upscaling of deworming efforts is a perhaps unlooked-for consequence of COVID, but one that looks set to ensure that the momentum and success of deworming programmes will continue.

## Data Availability

This article has no additional data.
